# Timely deposition of macromolecular structures is necessary for peer review

**DOI:** 10.1107/S0907444913024621

**Published:** 2013-11-19

**Authors:** Robbie P. Joosten, Hayssam Soueidan, Lodewyk F. A. Wessels, Anastassis Perrakis

**Affiliations:** a Biochemistry, Netherlands Cancer Institute, Plesmanlaan 121, 1066 CX Amsterdam, The Netherlands; b Molecular Carcinogenesis, Netherlands Cancer Institute, Plesmanlaan 121, 1066 CX, Amsterdam, The Netherlands

**Keywords:** Protein Data Bank, deposition, validation

## Abstract

Most of the macromolecular structures in the Protein Data Bank (PDB), which are used daily by thousands of educators and scientists alike, are determined by X-ray crystallography. It was examined whether the crystallographic models and data were deposited to the PDB at the same time as the publications that describe them were submitted for peer review. This condition is necessary to ensure pre-publication validation and the quality of the PDB public archive. It was found that a significant proportion of PDB entries were submitted to the PDB after peer review of the corresponding publication started, and many were only submitted after peer review had ended. It is argued that clear description of journal policies and effective policing is important for pre-publication validation, which is key in ensuring the quality of the PDB and of peer-reviewed literature.

## Abstract 2

Deposition of crystallographic structures should be concurrent with or prior to manuscript submission for peer review, enabling validation and increasing reliability of the PDB.

## 1. Introduction

Since the mid-1990s, peer-reviewed journals and the crystallographic community have worked towards the notion that crystallographic models and the associated diffraction data should be submitted to the Protein Data Bank (Baker *et al.*, 1996 ▶) and publicly released upon publication (Wlodawer *et al.*, 1998 ▶; Editorial, 1998 ▶; Baker & Saenger, 1999 ▶). This is nowadays the norm, and deviations from that rule are rare. As much as 99.8% of crystallographic structures submitted to the PDB within 2011–2013 make available both the model and the experimental data. This also enables critical re-evaluation of submitted models, based on the original diffraction data but in the light of improved methods and software (Joosten *et al.*, 2009 ▶). However, the time frame for data submission has been less well defined: should data be available in one of the wwPDB (Berman *et al.*, 2003 ▶) sites before the paper is submitted, before it is accepted for publication, or merely after the paper is accepted, just before publication?

Recently, a Validation Task Force assigned by the PDB has published a recommendation (Read *et al.*, 2011 ▶) that the submission of papers that report on crystallographic data should be accompanied by a validation report issued from the PDB. It is an obvious pre­requisite that both the experimental data and the model coordinates are submitted to the PDB before paper submission, to achieve this. Such reports are indispensable tools for technical review of the paper by the assigned referees (Read *et al.*, 2011 ▶), and crucial for ensuring that any claims based on the structure are supported by data of appropriate quality.

## 2. Materials and methods

The original data presented in this paper are available in public databases (PDB and PubMed); a data digest relevant to our conclusions are included as Supplementary Material;^1^ and all the code and the database as well as minimal instructions to reproduce all the results have been uploaded to GitHub, at the repository https://github.com/massyah/PdbMine.

Briefly, the identifier of PDB records with associated ‘Primary citation’ were retrieved from the RCSB webserver on 28 June 2013 at 15:25 GMT+1 (91 738 unique IDs). The corresponding PDB entries were downloaded from the ftp.wwpdb.org FTP server, parsed, and the PDB fields relevant for this study (namely PDB ID, date of deposition, associated PubMed ID) were stored in a SQLITE3 database. The PubMed entries of all associated citations were downloaded from the PubMed web server using the *EUTILS* suite and then parsed and stored in the SQLITE3 database. From the PubMed associated MEDLINE records, we extracted (if available) the following dates: received, revised, accepted and ahead of print date from the publication history (PHST) field; date of publication (DP); date created (DA); PubMed central release date (PMCR); date of electronic publication (DEP) and Entrez Date (EDAT). The ‘earliest public date’ is then defined as the earliest of the PubMed dates; while the ‘earliest publication date’ is defined as the earliest of the DP, EDAT, DA, DEP and the ‘ahead of print’, ‘accepted’ dates from the PHST. We then considered for this analysis the inner join of the PDB entries table with the PubMed table, where we only kept entries for which (i) the earliest public date was after 1 January 1995; (ii) the published date and accepted date were before 1 January 2014 or available; and (iii) either the publication history was available or the received date was earlier than the accepted or published date; totalling 69 026 unique PDB entries joined with 35 924 unique PubMed entries.

All entries were considered to be ‘on time’ by default. We defined as ‘deposited after acceptance’ those entries for which the date of deposition with the PDB was more than two days after the ‘earliest publication date’. We identified as ‘deposited after submission’ those entries that were not ‘deposited after acceptance’ but for which deposition with the PDB was more than two days after the ‘earliest public date’. The impact-factor estimates used to build Table 1 ▶ originate from the Thomsom Reuters Journal Citation Reports Science Edition 2011 (http://thomsonreuters.com/journal-citation-reports/).

## 3. Results and discussion

### 3.1. Correlating the dates of crystallographic structure and data submission to the PDB and of manuscript submission for peer review

**Figure 1:**
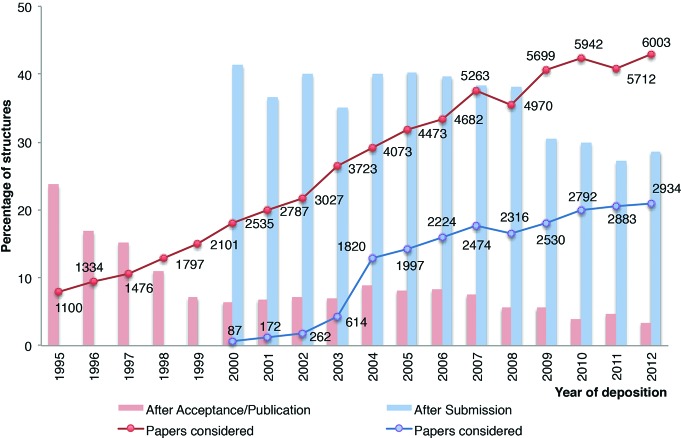
Deposition dates of structures during the different editorial phases of the corresponding manuscript. Red columns show the percentage of structures that were deposited after the manuscript was accepted (or after it was published if acceptance dates were not available) and blue columns show the percentage of structures deposited after the manuscript was submitted for review but before it was accepted/published. The lines show the number of manuscripts for which the appropriate editorial history was available for each of these categories. Note that before 2000 insufficient data were available on manuscript submission dates.

The results from the analysis of the PDB deposition date against the submission and acceptance dates were manually curated to select journals with at least 100 publications that referred to PDB entries over the last 12 years, and are presented in Table 1 ▶. The number of structures submitted to the PDB only after the paper was accepted for publication has historically been rather low (less than 10% since 1999) and has been minimized over the years, being just 3.4% (205 of 6003 papers) in 2012 (Fig. 1 ▶). However, the number of structures submitted to the PDB after the paper has been submitted for review is, somewhat surprisingly, high. Although tracing the submission date is not possible for all publications, we were able to extract that information for about 50% of the structures published in 2012, and about one third of them were deposited after the paper was submitted to the journal for peer review. It is also noteworthy, that a quarter of the depositions in the window between manuscript submission and manuscript acceptance occurred just within the last six days before manuscript acceptance (Supplementary Fig. S1). It is unlikely that referees had access to PDB validation reports in that time window, and more likely that formal acceptance of the manuscript was postponed until the structure was deposited.

### 3.2. Confidentiality *versus* transparency issues

Many authors are worried that submission of a structure to the PDB will trigger competitors to accelerate their own paper submission. This is a legitimate concern, and having been at the receiving end of this practice, this is not a pleasant experience. However, this concern is ameliorated by an existing submission-time option where the sequences corresponding to the submitted structures are not made publically available before the entry is finally released. The possibility of not directly disclosing the sequence is popular: it is currently used by about two thirds of entries awaiting release. A submission-time option to also withhold the title, currently only possible upon request, would undoubtedly prove equally popular and could help removing remaining concerns.

### 3.3. Some journals are more equal than others

Urban legend has it that high-impact journals are notorious for tolerating late submission as they typically publish ‘hot’ structures, which many research groups are competing to be the first to determine: to paraphrase a well known quotation (Orwell, 1945 ▶), all journals are equal, but some journals are more equal than others. Indeed, we find that journals with a high impact factor for which we could trace the full publication history (the list most regrettably does not include important journals like *Science*, *Proc. Natl Acad. Sci. USA* and *J. Biol. Chem.*, which do not make the complete publication history available in the PubMed/MEDLINE records) are more likely to tolerate late submission of crystallographic data (Supplementary Fig. S2). A notable exception to this rule is *Acta Crystallographica Section D*, which traditionally had a significantly lower impact factor (between 1 and 3) and has only shot to impact-factor prominence over the last couple of years (mainly owing to the publication of highly cited methodological papers). One of the best performing journals in recent years is *Proteins*, which unsurprisingly has a simple, clear and short policy statement in the instruction for authors: ‘For all crystallographic studies, coordinates and structure factors should be deposited in the Protein Data Bank at the time of manuscript submission’. This policy, unlike others (a survey of the policies of different journals is available as Supplementary Table S1) is explicit about the timing of deposition. Clarity about policies is crucial, but ensuring that the policies are honored is key.

## 4. Conclusion

As we are confident that all journals strive for transparency in the publication procedure and for rigor in the reported results, we strongly advocate that the editorial teams improve the clarity of their policies, and enforce these effectively. The structural biologists, authors and reviewers alike, should also share the responsibility for following these policies. As a community we must strive to ensure that coordinates and experimental data for macromolecular models are submitted to the PDB at the same time as the paper is submitted for review. Only then will validation reports also become available to the referees as part of the necessary material for peer review.

## Supplementary Material

Supplementary material file. DOI: 10.1107/S0907444913024621/dz5303sup1.pdf

## Figures and Tables

**Table 1:** Numbers and percentages of papers for which the associated PDB entries were submitted after the submission date or after the acceptance or publication date, per journal and associated journal impact factors (IF), for journals for which data were available for more than 100 structures for the period between 2000 and 2012

			Deposition date with PDB after				
	No. of		Submission		Acceptance†		
Journal	Structures	Papers	No.	%	No.	%	IF (2011)
*J. Mol. Biol.*	8885	5467	1074	20	622	7	4.0
*Structure*	3501	2045	813	40	408	12	6.3
*Acta Cryst. D*	2688	2310	545	24	154	6	12.6
*Nature Struct. Mol. Biol.*	2525	1445	864	60	226	9	12.7
*Nature (London)*	1966	1476	1020	69	244	12	36.2
*Protein Sci.*	1907	215	18	8	103	5	2.8
*EMBO J.*	1826	1061	543	51	228	12	9.2
*Proteins*	1588	166	9	5	28	2	3.3
*Bioorg. Med. Chem. Lett.*	1348	1299	732	56	93	7	2.5
*Cell*	1147	711	471	66	138	12	32.4
*Mol. Cell*	1084	788	554	70	115	11	14.2
*PLoS One*	779	779	146	19	42	5	4.1
*Acta Cryst. F*	665	665	60	9	30	5	0.5
*Biochem. J.*	590	61	21	34	41	7	4.9
*FEBS J.*	549	42	3	7	19	3	3.8
*J. Struct. Biol.*	537	495	75	15	35	7	3.4
*Biochem. Biophys. Res. Commun*.	484	417	18	4	51	11	2.5
*FEBS Lett.*	469	302	51	17	81	17	3.5
*Chem. Biol.*	461	338	114	34	120	26	5.8
*Angew. Chem. Int. Ed. Engl.*	353	128	43	34	20	6	13.5
*Nature Chem. Biol.*	351	348	184	53	28	8	14.7
*Biochim. Biophys. Acta*	331	277	46	17	21	6	3.6
*PLoS Pathog.*	262	262	89	34	39	15	9.1
*Bioorg. Med. Chem.*	254	239	48	20	26	10	2.6
*Chembiochem*	242	106	16	15	8	3	3.9
*J. Biol. Inorg. Chem.*	203	171	37	22	17	8	3.3
*Biophys. J.*	196	51	7	14	22	11	3.6
*PLoS Biol.*	185	185	84	45	18	10	11.5
*J. Biomol. NMR*	181	87	12	14	28	15	3.6
*BMC Struct. Biol.*	176	176	34	19	7	4	2.5
*Arch. Biochem. Biophys.*	167	142	16	11	7	4	2.9
*ChemMedChem*	158	74	6	8	2	1	3.2
*EMBO Rep.*	153	147	67	46	22	14	7.4
*Immunity*	150	94	30	32	20	37	21.6
*J. Struct. Funct. Genomics*	131	115	6	5	5	1	n/a
*Nature Commun.*	119	119	59	50	50	2	7.3
*J. Inorg. Biochem.*	101	79	20	25	20	6	3.0

† Or publication, if the submission date is not available.

## Footnotes

1 Supplementary material has been deposited in the IUCr electronic archive (Reference: DZ5303). Services for accessing this material are described at the back of the journal.
