# Supplementary material for: Timely deposition of macromolecular structures is necessary for peer review
Source: Acta Crystallogr D Biol Crystallogr. 2013 Nov 19;69(Pt 12):2293–5. doi: 10.1107/S0907444913024621 (PMC3852646; doi:10.1107/S0907444913024621)
Supplement: Supplementary file 1 [file d-69-02293-sup1.pdf]

Supplementary Material

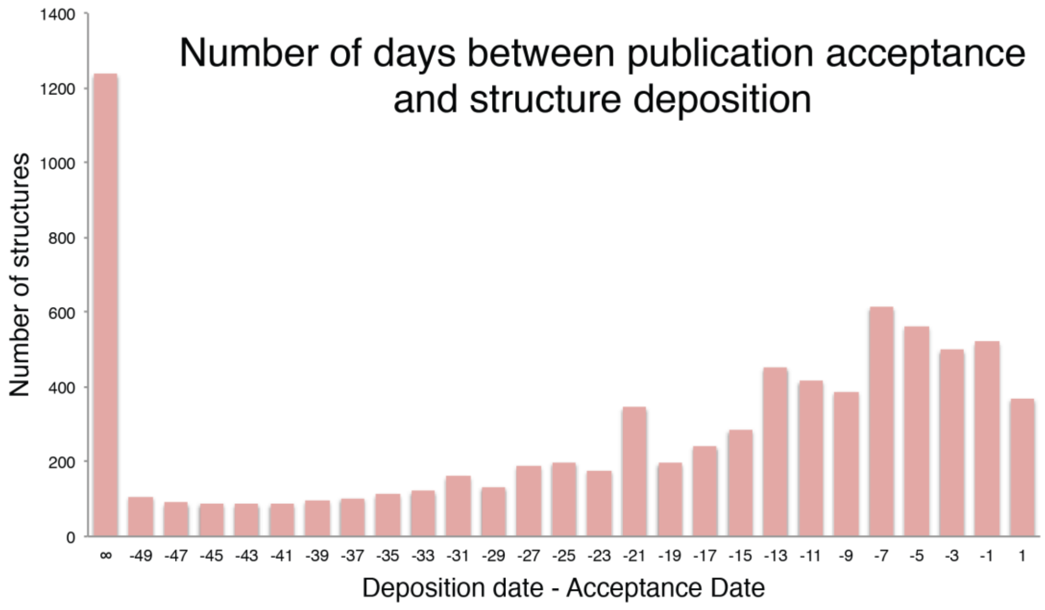

Figure S1. Histogram of the difference between the PDB deposition date and the manuscript acceptance date.

High Impact

Journal	2002	2003	2004	2005	2006	2007	2008	2009	2010	2011	2012
Nature	16/78	52/68	68/79	110/130	69/103	114/149	130/179	76/115	123/176	120/148	153/188
Cell	1/50	1/53	55/81	70/78	65/86	69/82	35/64	49/57	47/67	83/91	47/95
Cancer Cell			1/1	3/3	1/13	0/2	2/2	1/1	3/3	3/7	2/2
Nat Immunol	2/4	5/8		4/8	4/6	4/10	1/5	4/4	3/3	12/12	3/3
Nat Biotechnol	3/3		8/9	2/2	2/2	0/2		1/7	1/1	3/3	1/12
Immunity	0/8	0/5	0/4	0/1	1/5	15/19	5/12	19/24	1/1	16/16	12/17
Nat Chem								0/5	0/7	0/8	6/23
Neuron	0/3	3/5	4/5	0/9	2/2	6/8	0/6		0/7		
Nat Chem Biol				3/3	10/28	7/22	42/56	41/57	26/39	43/67	18/39
Mol Cell	5/52	14/87	75/104	60/89	84/109	113/139	59/80	63/79	53/70	44/49	47/63
Dev Cell	0/1	0/1	1/1	2/2	2/2	4/5	5/8	1/1	4/4	1/2	5/5
Angew Chem Int Ed Engl	0/4	1/6	2/7	0/29	8/28	3/20	0/17	2/60	0/43	7/37	36/72
Nat Struct Mol Biol	31/146	95/177	91/126	57/103	76/94	95/117	57/105	125/165	154/225	87/116	109/151
Acta Crystallogr D	79/168	51/173	57/192	91/212	73/211	61/182	47/159	20/157	27/155	9/166	14/199
PLoS Biol		2/2	12/14	16/19	21/21	5/16	6/9	13/18	13/30	7/34	2/16
EMBO J	6/136	3/170	80/123	76/114	140/189	55/96	60/102	68/130	41/84	80/118	38/49
PLoS Pathog				1/1		7/9	20/21	9/17	14/39	36/64	31/86
Nat Commun									7/7	14/26	15/51
EMBO Rep	0/2	3/5	2/11	6/11	2/8	18/30	11/19	13/23	8/10	13/19	8/8
Structure	6/200	49/269	136/275	141/234	82/135	141/226	112/215	112/210	90/175	90/189	112/281
Chemistry	0/1	1/1	0/1	0/3	0/3		0/7	2/23	1/15	0/17	1/15
Chem Biol	2/22	5/28	37/49	19/24	15/21	15/27	33/55	18/31	20/39	30/43	26/41
Biochem J	1/13	19/29	6/30	1/32	7/46	5/45	9/58	9/48	2/82	1/85	1/55
PLoS One						10/14	2/21	25/67	23/93	62/206	56/276
J Mol Biol	12/473	31/601	261/628	236/617	310/730	281/798	247/711	113/685	40/579	10/422	7/228
Chembiochem	0/6	0/11	1/5	1/16	2/13	2/17	1/17	1/23	0/21	5/38	1/44
FEBS J				0/67	0/48	6/71	11/57	1/69	0/70	0/64	4/90
Biophys J	0/4	2/15	0/7	3/9	2/33	0/12	2/22	2/18	4/14	4/5	0/6
Biochim Biophys Acta	0/10	4/14	2/11	9/15	12/18	4/20	5/36	6/36	13/41	1/45	7/50
J Biomol NMR	2/15	0/10	1/19	2/15	1/11	1/10	2/10	1/7	4/10	2/5	0/16
FEBS Lett	1/19	0/31	16/38	15/33	5/22	7/31	9/35	13/33	7/45	12/38	5/26
Appl Microbiol Biotechnol										2/12	3/8
J Struct Biol	10/17	0/6	17/23	3/28	11/28	6/28	5/25	6/65	26/110	6/80	16/97
Proteins	2/56	0/45	1/121	1/192	6/136	4/205	1/198	1/150	5/111	0/63	3/46
J Inorg Biochem	2/6	0/3	5/6	0/2	1/8	1/9	1/3	4/5	4/4	2/3	5/36
Virology	0/1	1/9	1/1	3/4	0/1	0/6	4/5	4/9	1/5	4/4	3/6
Eur J Med Chem		1/1				0/2	0/7	1/9	0/4	0/6	1/19
J Biol Inorg Chem	4/12	6/9	6/13	8/15	5/20	6/12	0/11	1/17	5/25	0/16	2/8
ChemMedChem					0/17	1/10	0/4	1/27	0/23	4/43	1/25
Biochimie	0/1		1/1	1/5	0/4	0/1	0/1	0/2	0/15	0/12	4/19
Arch Biochem Biophys	0/5	1/8	0/1	0/12	3/5	8/31	0/12	2/11	5/17	0/29	2/17
Mol Immunol						5/7	0/2	1/7	0/7	0/9	0/3
Protein Sci	1/66	6/104	1/110	7/140	1/100	4/157	2/125	1/155	5/102	18/123	10/90
J Synchrotron Radiat		4/4		0/2	8/8	1/2	6/6		1/3	7/9	
Bioorg Med Chem Lett	0/3	2/35	28/41	75/101	83/118	109/134	100/157	101/171	121/216	92/162	81/134
Bioorg Med Chem	1/8	0/3	2/5	8/19	7/16	10/14	11/27	6/30	12/58	9/32	3/23
Mol Biochem Parasitol		0/2		0/5	8/33	0/4	0/1	0/6	0/1	0/6	0/3
Biochem Biophys Res Commun	1/17	1/24	5/36	13/34	4/43	6/51	4/32	19/74	7/60	1/42	2/38
BMC Struct Biol	2/2	0/5	0/4	2/3	4/7	14/61	6/21	4/29	5/13	3/16	0/14
Int J Biol Macromol	1/1						0/4		0/10	2/7	1/6
Cell Rep											18/23
Bioorg Chem	0/3			0/2	9/10	0/2		0/1	0/5		
Acta Crystallogr F			3/9	6/37	14/70	9/55	13/59	16/99	10/98	9/127	7/80
Protein Cell									6/13	3/11	4/9
J Struct Funct Genomics		0/12	0/7	0/5		0/7	1/8	0/4	4/46	1/27	1/15

Low Impact

Bad neutral Good

After Submission / Total

Figure S2. "Heatmap" type table showing the number of structures deposited after the manuscript was submitted for peer review, as a function of the publication year, for journals that publish a significant number of structures ordered by their 2011 ISI impact factor.

Table S1: editorial policies on model and data deposition for macromolecular X-ray structures

Data retrieved on July 2nd 2013

Journal	Policy summary
Acta Crystallogr. D	Authors should follow the deposition recommendations of the IUCr Commission on Biological Macromolecules (...). For all structural studies of macromolecules, coordinates and the related experimental data (...) must be deposited at a member site of the [wwPDB][1] (...) if a total molecular structure has been reported. Authors are encouraged to deposit their data with the wwPDB in advance of submission to the journal and to provide an mmCIF and a wwPDB validation report which will be required on submission. (...) Authors must supply the wwPDB reference codes before the article can be published and the data must be released upon publication.
Acta Crystallogr. F	Authors should follow the deposition recommendations of the IUCr Commission on Biological Macromolecules (...). For all structural studies of macromolecules, coordinates and the related experimental data (...) must be deposited at a member site of the [wwPDB] (...) if a total molecular structure has been reported. Authors are encouraged to deposit their data with the wwPDB in advance of submission to the journal and to provide an mmCIF and a wwPDB validation report which will be required on submission. Authors must supply the wwPDB reference codes before the article can be published and the data must be released upon publication.
Biochemistry	For papers describing structures of biological macromolecules, the atomic coordinates and the related experimental data (...) must be deposited at a member site of the [wwPDB] (...). The PDB ID should be included in the manuscript. Authors must agree to release the atomic coordinates and experimental data when the associated article is published. (...) A manuscript will be accepted only after receipt from the submitting author of a written statement that the coordinates have been deposited. Coordinates must be released immediately upon publication.
Bioorg. Med. Chem. Lett.	*No public policy on deposition of model and experimental data*
Cell	For papers describing structures of biological macromolecules, the atomic coordinates and related experimental data (...) must be deposited at a member site of the [wwPDB]. (...) The corresponding database IDs must be included in the manuscript. Authors must agree to release atomic coordinates and experimental data when the associated article is published. Additionally, Cell now recommends that the authors include PDB validation report as a part of the Supplemental Information for all new submissions describing results of X-ray and NMR structure determination.
EMBO J.	The EMBO Journal accepts and follows the recommendations of the International Union of Crystallography (IUCr), with regard to the deposition and release of macromolecular structural data. (...) In summary, they state that all publications must be accompanied by deposition of both the atomic coordinates and the structure-factor amplitudes in the appropriate database (PDB or NDB). In the case of low-resolution structures for which only a chain trace is reported, a set of C alpha positions and structure-factor amplitudes may be sufficient.
J. Am. Chem. Soc.	Any set of atomic coordinates referred to in the manuscript, including atomic coordinates and structure factors for proteins determined by X-ray crystallography and coordinates determined by NMR, should be deposited with the Protein Data Bank (...) whenever appropriate. (...) If the coordinate files are not deposited in the PDB, or if the PDB files are on hold until publication, then the coordinate files must be included in the Supporting Information submitted concurrently with the manuscript. (...) A manuscript that does not provide coordinates at the time of submission will not be sent out for review. It is the responsibility of the author to obtain a file name (PDB ID or NDB ID) for the molecule; the file name must appear in the published manuscript. If a file name has not yet been obtained upon acceptance of a paper, it must be added in proof. Atomic coordinates and structure factors for all structures mentioned must be available immediately upon publication of the paper, either directly in Supporting Information, or as a data bank deposition.
J. Biol. Chem.	For papers describing structures of biological macromolecules, the atomic coordinates and the related experimental data (...) must be deposited at a member site of [wwPDB] (...) The PDB ID assigned after deposition should be included in the manuscript. Authors must also submit the PDB Summary Validation Report (provided after annotation by the wwPDB) to JBC for review at the time of submission. PDB data must be ready for release before final acceptance of the manuscript. No data are to be withdrawn from PDB once a paper has been accepted and published as a Paper in Press article.

J. Med. Chem.	For papers describing structures of biological macromolecules, the atomic coordinates and the related experimental data (...) must be deposited at a member site of the [wwPDB](...) The PDB ID must appear before the references (...) Authors must agree to release the atomic coordinates and experimental data when the associated article is published.
J. Mol. Biol.	For papers describing structures of biological macromolecules, the atomic coordinates and the related experimental data (...) must be deposited at a member site of the [wwPDB] (...) Manuscripts must carry a statement that coordinates and structure factors (...) have been deposited in the Protein Data Bank. The accession number(s) must be cited in the manuscript at the end of the Materials and Methods section. Authors must agree to release the atomic coordinates and experimental data immediately upon publication.
Mol. Cell	For papers describing structures of biological macromolecules, the atomic coordinates and related experimental data (...) must be deposited at a member site of the [wwPDB](...) The corresponding database IDs must be included in the manuscript. Authors must agree to release atomic coordinates and experimental data when the associated article is published. Additionally, Molecular Cell now recommends that the authors include the PDB validation report as a part of the Supplemental Information for all new submissions describing results of X-ray and NMR structure determination.
Nat. Struct. Mol. Biol. (formerly Nat. Struct. Biol.)	Authors of papers describing structures of biological macromolecules must provide atomic coordinates and related experimental data (...) upon request of editors for the purposes of evaluating the manuscript, if they are not already freely accessible in a publicly available and recognized database (for example, Protein DataBank) (...). Accessibility must be designated "for immediate release on publication".
Nature	Authors of papers describing structures of biological macromolecules must provide atomic coordinates and related experimental data (...) upon request of editors for the purposes of evaluating the manuscript, if they are not already freely accessible in a publicly available and recognized database (for example, Protein DataBank) (...) Accessibility must be designated "for immediate release on publication". For papers published in Nature, a weekly publication with a twice-weekly AOP publication schedule, the author must authorize Protein DataBank (PDB) release on the Wednesday of (or before) online or print publication. (The journal office informs authors of the publication date as soon as articles are scheduled, and also informs the PDB directly.)
Nucleic Acids Res.	Sequence information, co-ordinates used to create molecular models described in a manuscript, and structural data must be submitted in electronic form, prior to acceptance, to the appropriate database for release no later than the date of publication of the corresponding article in the Journal. Deposition numbers and/or accession numbers provided by the database should be included in the manuscript and entered into the relevant boxes during online submission or communicated to the Executive Editor handling the manuscript as soon as received (...). Atomic co-ordinates may be included in the publication as supplementary material. Manuscripts will not be published until the Journal is in receipt of the deposition number. (...)<br>For papers reporting novel three-dimensional structures<br>Atomic co-ordinates and the related experimental data (...) must be deposited with a database. Authors must agree to release the atomic coordinates and experimental data when the associated article is published.
Proc. Natl. Acad. Sci. USA	For papers describing structures of biological macromolecules and small molecules, the atomic coordinates and the related experimental data (...) must be deposited at a member site of the [wwPDB] (...). The PDB ID should be included in the manuscript. (...) Authors must agree to release the atomic coordinates and experimental data when the associated article is published.
Protein Sci.	Structure Coordinates. For papers describing structures of biological macromolecules, the atomic coordinates and the related experimental data (...) must be deposited at a member site of the [wwPDB] (...) The PDB ID should be included in the manuscript. Authors must agree to release the atomic coordinates and experimental data when the associated article is published.
Proteins	Please note that for all reports of new crystallographic structures, coordinates and structure factors should be deposited in the Protein Data Bank at the time of manuscript submission. The PDB id(s) should be included in the text. These data must be released upon publication of the accepted article (...). Authors should note that the PROTEINS Editorial Board encourages release of coordinates and structure factors at the time of PDB deposition. Coordinates and structure factors might be requested from authors as part of the review process.
Science	Atomic coordinates and structure factor files from x-ray structural studies or an ensemble of atomic coordinates from NMR structural studies must be deposited and released at the time of publication. Approved databases are the [wwPDB] (...).

Structure	For papers describing structures of biological macromolecules, the atomic coordinates and related experimental data (...) must be deposited at a member site of the [wwPDB] (...) The corresponding database IDs must be included in the manuscript. Authors must agree to release atomic coordinates and experimental data when the associated article is published. Additionally, Structure now recommends that the authors include PDB validation report as a part of the supplemental information for all new submission describing results of X-ray and NMR structure determination.

[1] Worldwide Protein Data Bank; all occurrences are abbreviated to ‘wwPDB’
